# TP-ARMS: A Cost-Effective PCR-Based Genotyping System for Precision Breeding of Small InDels in Crops

**DOI:** 10.3390/ijms27031406

**Published:** 2026-01-30

**Authors:** Yuan Wang, Jiahong Chen, Yi Liu

**Affiliations:** 1 School of Biological Science and Technology, University of Jinan, Jinan 250022, China; yuanwang@psc.ac.cn; 2 Key Laboratory of Grain Crop Genetic Resources Evaluation and Utilization, Ministry of Agriculture and Rural Affairs, Shanghai Agrobiological Gene Center, Shanghai 201106, China

**Keywords:** small insertion/deletion (InDel), genotyping, TP-ARMS, rice (*Oryza sativa*), marker-assisted breeding

## Abstract

Accurate genotyping of small insertions and deletions (InDels; <5 bp) remains technically challenging in routine molecular breeding, largely due to the limited resolution of agarose gel electrophoresis and the labor-intensive nature of polyacrylamide-based assays. Here, we present the Tri-Primer Amplification Refractory Mutation System (TP-ARMS), a simple and cost-effective PCR-based strategy that enables high-resolution genotyping of small InDels using standard agarose gels. The TP-ARMS employs a universal reverse primer in combination with two allele-specific forward primers targeting insertion and deletion alleles, respectively. This design allows clear discrimination of homozygous and heterozygous genotypes using a two-tube PCR workflow. The method showed complete concordance with Sanger sequencing in detecting 1–5 bp InDels across multiple crop species, including rice (*Oryza sativa*) and quinoa (*Chenopodium quinoa*). In addition, using a TP-ARMS reduced experimental time by approximately 90% compared with PAGE-based approaches and avoided the high equipment and DNA quality requirements of fluorescence-based assays. The practical applicability of the TP-ARMS was demonstrated in breeding populations, including efficient genotyping of a 3-bp InDel in *OsNRAMP5* associated with cadmium accumulation and a 6-bp promoter InDel in *OsSPL10* underlying natural variation in rice trichome density across 370 accessions. Collectively, the TP-ARMS provides a robust, scalable, and low-cost solution for precise small InDel genotyping, with broad applicability in marker-assisted breeding and functional genetic studies.

## 1. Introduction

The rapid expansion of genomic and pan-genomic resources has substantially accelerated the identification of functional genetic variations underlying agronomic traits in crops [1,2,3]. Large-scale sequencing efforts in both major and minor crops, including rice and quinoa, have revealed millions of polymorphisms distributed across both coding and regulatory regions [4,5]. For instance, the 3000 Rice Genomes Project has systematically cataloged extensive genetic variants, including single nucleotide polymorphisms (SNPs) and insertions/deletions (InDels), many of which are associated with critical agronomic traits such as yield potential and disease resistance [6]. With the rapid accumulation of genomic data, leveraging these resources effectively has become a pressing challenge. In this context, molecular markers have emerged as essential tools for precision breeding. They enable efficient selection of traits such as drought tolerance, pathogen resistance, and have become integral to modern agricultural improvement programs [7].

Small InDels (1–5 bp) are particularly valuable for marker development due to their high abundance in plant genomes compared to larger InDels, SNPs, and structural variants, as well as their potential to directly influence gene expression and protein function. In addition, their polymorphic nature makes them ideal for high-resolution genotyping across diverse populations [8]. Despite these advantages, conventional InDel genotyping suffers from notable technical limitations. Sanger sequencing, while highly accurate, remains cost-prohibitive for large-scale applications, with bidirectional sequencing typically approximately $2–3 per sample [9]. Agarose gel electrophoresis, a routine technique in molecular biology, lacks sufficient resolving power to reliably distinguish InDels smaller than 5 bp because it relies primarily on fragment length rather than sequence specificity. Polyacrylamide gel electrophoresis (PAGE) provides higher resolution and enables the detection of 1–5 bp differences. However, PAGE is labor-intensive and involves toxic acrylamide, prolonged electrophoresis times (>4 h), and specialized staining procedures, which limit its suitability for high-throughput genotyping [10,11].

Recent innovations, such as Kompetitive Allele-Specific PCR (KASP), have enabled high-throughput SNP and InDel genotyping [12]. However, KASP’s dependence on fluorescent probes and costly real-time PCR systems, often exceeding $50,000, limits its accessibility in resource-constrained laboratories [13]. Additionally, KASP typically requires high-purity DNA templates (A260/A280 > 1.8), a criterion that is frequently difficult to meet when working with field-collected samples or large breeding populations [14]. Collectively, these constraints underscore the urgent need for a cost-effective, high-resolution genotyping strategy that is practical for broad implementation in molecular breeding programs [15].

To address these gaps, we developed the Tri-Primer Amplification Refractory Mutation System (TP-ARMS), a PCR-based approach optimized for rapid, affordable detection of small InDels. Building on the conventional Amplification Refractory Mutation System (ARMS) [16], TP-ARMS incorporates two key design features. First, a shared reverse primer targeting conserved regions flanking the InDel reduces primer synthesis costs by 25% compared to traditional ARMS, which requires four primers per locus [17]. Second, two allele-specific forward primers with 3′-terminal mismatches selectively amplify wild-type or mutant alleles, enabling clear differentiation of heterozygotes in a two-tube workflow.

TP-ARMS enables single-base resolution using standard 0.75–1% agarose gels, thereby eliminating the need for PAGE or capillary electrophoresis. In proof-of-concept trials, use of the TP-ARMS accurately genotyped a 1-bp InDel in *OsERF65*, a rice gene regulating sheath blight resistance. The results showed 100% concordance with Sanger sequencing and outperformed conventional PCR-based assays. Its compatibility with crude DNA extracts (A260/A280 ≥ 1.5) and basic thermocyclers makes the TP-ARMS a transformative tool for molecular breeding, especially in developing regions with limited infrastructure. By providing accessible, high-precision genotyping, the TP-ARMS can facilitate the development of climate-resilient crop varieties. This approach supports the practical application of genomic resources in modern breeding programs.

## 2. Results

**Figure 1:**
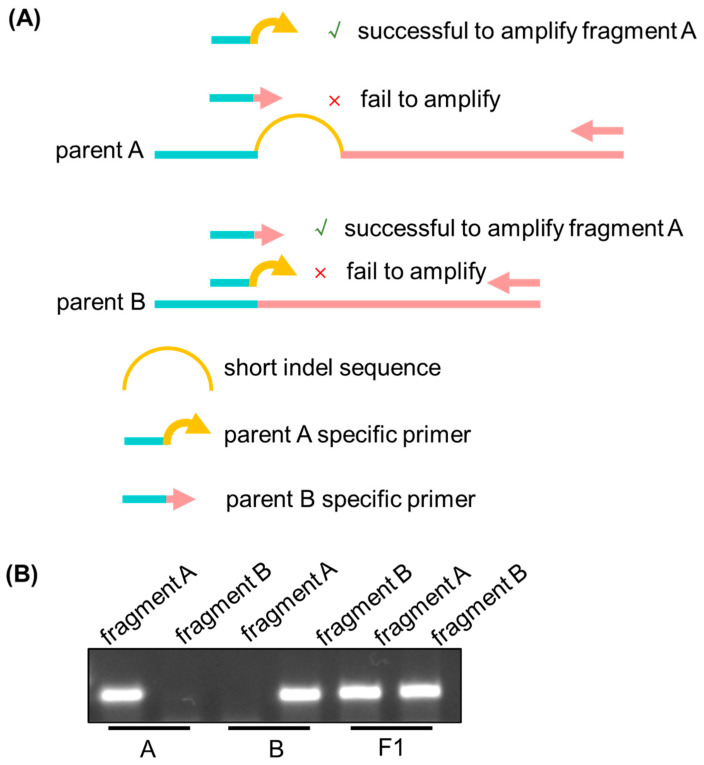
The principle of TP-ARMS. (**A**) Schematic diagram of Tri-AMRS. (**B**) PCR bands of parents A and B and F1 by agarose gel electrophoresis under two sets of amplification primer systems. Parent A contained the insert sequence, so the target band was specifically amplified with insertion-F and universal R-primer. However, no bands could be amplified with deletion-F and universal R-primer. However, parent B does not contain the insert sequence, so it is the opposite of A. However, since the F1 generation is heterozygous individuals, bands could be amplified using both insertion-F and universal R-primer, as well as deletion-F and universal R-primer. Therefore, the system was able to distinguish between two homozygous parents and heterozygotes.

As shown in Figure 1A, Parent A and Parent B differ by a 1–5 bp insertion/deletion (InDel) within the target genomic region. To distinguish these alleles, we designed insertion-F and deletion-F primers that specifically target Parent A’s insertion allele and Parent B’s deletion allele, respectively. In addition, a universal reverse primer was also designed in a conserved region shared by both parental sequences. The combination of insertion-F and the universal reverse primer amplifies a DNA fragment exclusively in Parent A. No amplification was detected in Parent B. Conversely, the deletion-F and universal reverse primer pair selectively amplified DNA from Parent B. In hybrid F1 individuals (heterozygous for the InDel), both primer pairs generate distinct amplicons corresponding to Parent A and Parent B alleles (Figure 1A). By performing a two-tube PCR followed by agarose gel electrophoresis (0.8–1%), genotyping results are readily visualized in Figure 1B. Parent A exhibited a single band corresponding to the insertion-F–derived amplicon, whereas Parent B displayed a single band generated by deletion-F amplification. In contrast, F1 hybrids showed two distinct bands, consistent with their heterozygous genotype. Collectively, these results demonstrate that the TP-ARMS enables rapid and reliable discrimination of parental and hybrid genotypes using standard agarose gel electrophoresis.

**Figure 2:**
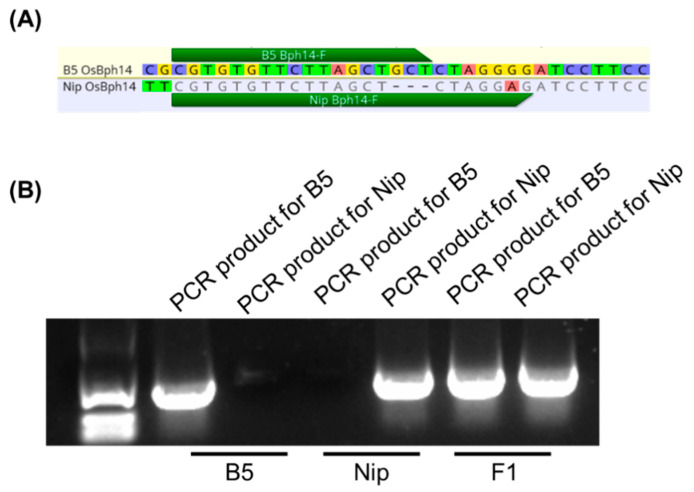
A 3-bp indel in B5 and Nip was distinguished by the TP-ARMS. (**A**) B5 and Nip present a 3-bp indel on the *OsBph14* gene region. (**B**) B5 contained the insert sequence, so the target band was specifically amplified with B5 *OsBph14*-F and *OsBph14*-R. However, no bands could be amplified with Nip *OsBph14*-F and *OsBph14*-R in B5. However, Nip does not contain the insert sequence, so it is the opposite of B5. However, since the F1 generation is heterozygous individuals, bands could be amplified using both B5 *OsBph14*-F and *OsBph14*-R, as well as Nip *OsBph14*-F and *OsBph14*-R. Therefore, the system was able to distinguish between B5, Nip, and heterozygous F1.

To evaluate whether our designed primers targeting small InDels can effectively discriminate short InDels in plant genomes, we initially conducted experiments in rice (*Oryza sativa*). The *OsBph14* is a key determinant of brown planthopper (BPH) resistance [18]. The rice cultivar B5 exhibits strong BPH resistance [19], whereas Nipponbare (Nip) is susceptible. Sequence comparison revealed a 3-bp InDel in the promoter region of OsBph14 between B5 and Nip (Figure 2A).

We designed allele-specific forward primers for B5 and Nip flanking this InDel, along with a universal reverse primer in a conserved region shared by both cultivars. Using two primer pairs (B5-specific forward and universal reverse; Nip-specific forward and universal reverse), we performed PCR amplification on B5, Nip, and their F1 hybrids. Distinct amplification patterns were observed among B5, Nip, and F1 individuals (Figure 2B). F1 hybrids displayed both parental bands, consistent with their heterozygous genotype.

The development of low-cadmium rice varieties holds critical significance for en-suring food safety and security. OsNRAMP5 has been identified as a key genetic target for regulating cadmium accumulation in rice. To advance low-cadmium rice breeding, we conducted a backcross between the low-cadmium cultivar *Shaoxiang 100* (carrying a 3-bp deletion in exon 11 of *OsNRAMP5*) and the conventional cultivar *Huhan1516*, generating a BC1F2 population (Supplementary Figure S1A). To validate the success of this genetic introgression, we performed cost-efficient genotyping of BC1F2 plants using the TP-ARMS. This method effectively distinguished the *OsNRAMP5* genotypes in BC1F2 individuals (Supplementary Figure S1B), overcoming the limitations of traditional Sanger sequencing for detecting small indels (e.g., the 3-bp deletion in *Shaoxiang 100*). These results demonstrate that the TP-ARMS can be effectively applied for genotyping small InDels in breeding populations, providing a practical strategy for large-scale molecular screening in cadmium-related rice breeding programs.

The InDels tested in the TP-ARMS described above were limited to 3–5 bp. To further evaluate the resolution limits of our TP-ARMS method, we investigated its ability to detect a 1-bp InDel in rice (*Oryza sativa*) and compared its performance with that of conventional PCR-based genotyping approaches. *OsERF65* has been reported to enhance rice resistance to sheath blight by improving reactive oxygen species (ROS) scavenging capacity. Using CRISPR-Cas9, we generated an edited OsERF65 line and identified an F1 individual carrying a 1-bp deletion in exon 2 of this gene through sequencing.

**Figure 3:**
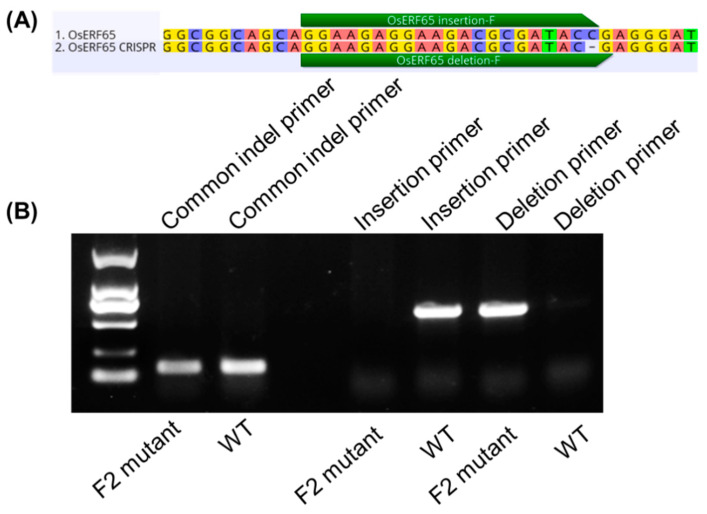
A 1-bp indel in *OsERF65* was distinguished by the TP-ARMS. (**A**) The wild-type rice and gene-editing F2 plants show 1-bp indel variation in *OsERF65*. (**B**) Conventionally designed indel primers could not distinguish wild-type plants with mutant F2 plants, but our Tri-ARMS primers were able to distinguish wild-type plants from mutant F2 plants.

Both TP-ARMS and conventional PCR primers flanking the InDel site were employed for genotyping analysis. Under 1% agarose gel electrophoresis conditions, the conventional method failed to distinguish between wild-type and mutant alleles due to negligible size differences. In contrast, the TP-ARMS successfully verified the 1-bp InDel, yielding distinct banding patterns (Figure 3 and Supplementary Figure S2). These results demonstrate the superior resolution of the TP-ARMS for detecting InDels smaller than 5 bp, highlighting its significant advantage over traditional approaches in precision genotyping.

To further validate the cross-species applicability of the Tri-Primer Amplification Refractory Mutation System (TP-ARMS), we conducted validation experiments in quinoa (*Chenopodium quinoa*). Epidermal bladder cells (EBCs) in quinoa are specialized structures that sequester excess sodium and potassium ions and play a critical role in salt tolerance [20,21]. Notably, we discovered a 5-bp InDel in the chromosome 8 between two quinoa varieties, Faro and QQ65, which exhibit contrasting EBC densities.

**Figure 4:**
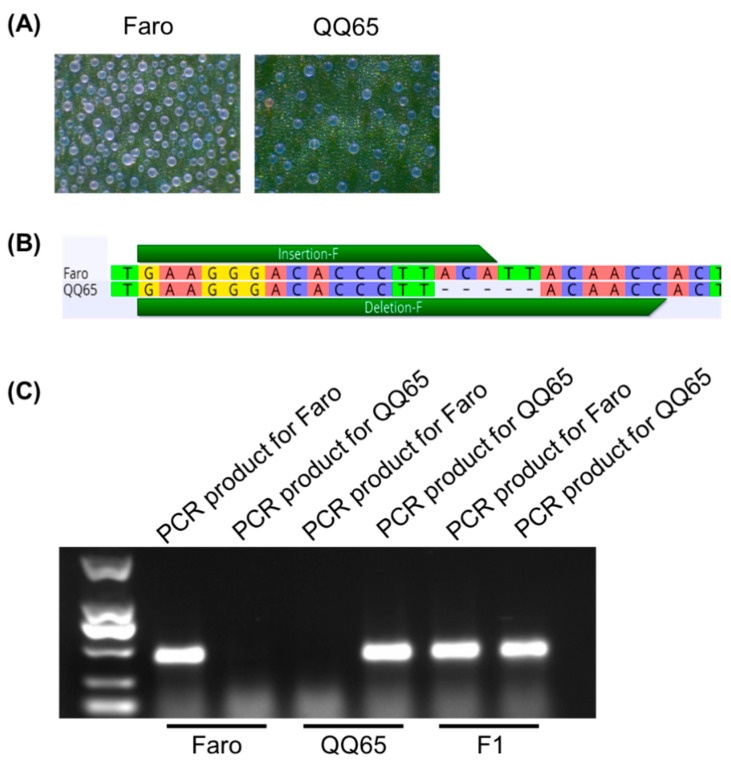
A 5-bp indel in Faro and QQ65 was distinguished by the TP-ARMS. (**A**) The leaf EBC phenotype of Faro and QQ65. (**B**) Faro and QQ65 showed a 5-bp indel in the promoter region of *CqREBCL1*. (**C**) The genotypes of Faro, QQ65, and the F1 were distinguished by the TP-ARMS.

Using the TP-ARMS, we successfully distinguished Faro, QQ65, and their F1 hybrids through allele-specific amplification followed by agarose gel electrophoresis (Figure 4). Faro produced a single amplicon generated by the Faro-specific primer pair, whereas QQ65 yielded a distinct band amplified by the QQ65-specific primer pair. F1 individuals displayed amplification products corresponding to both parental alleles, confirming their heterozygous genotype.

**Figure 5:**
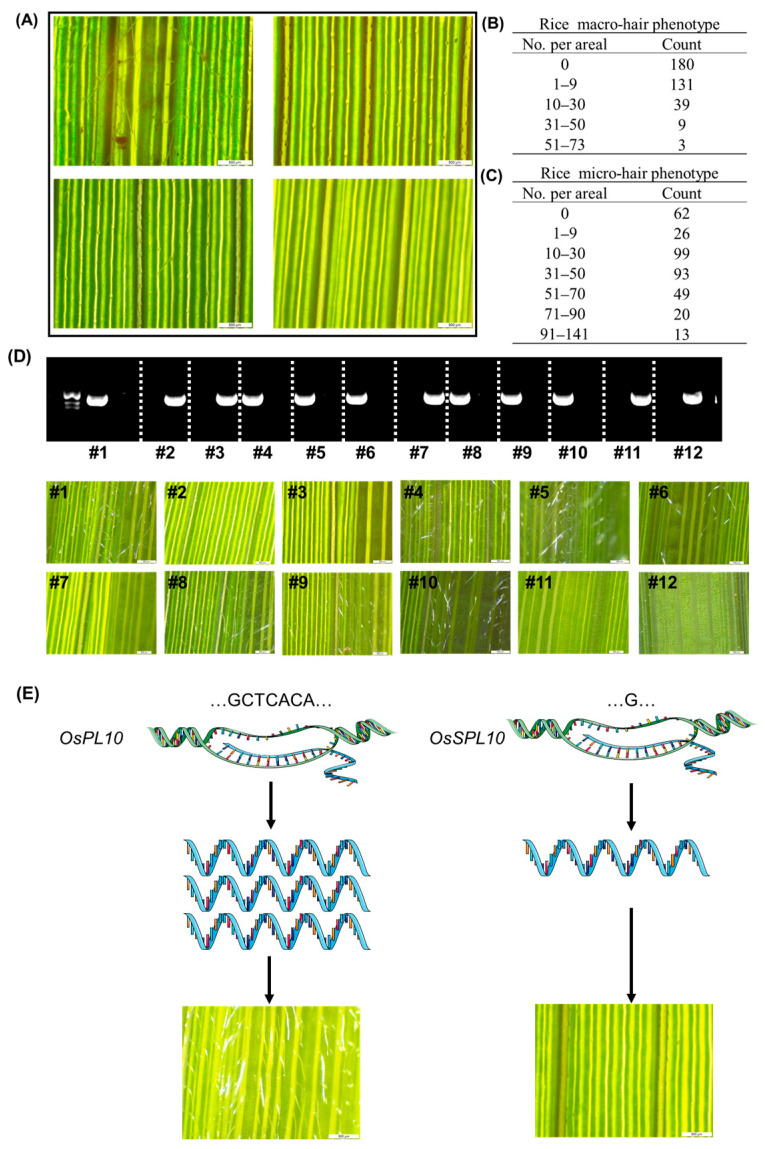
TP-ARMS identified an indel in the promoter region of *OsSPL10*. (**A**) The trichome phenotype of rice flag leaf. According to the type and number of hairs on the abaxial surface of the leaf, it can be divided into four types: leaves full of macro-hair, leaves with fewer macro-hairs and more micro-hairs, leaves full of micro-hairs, leaves that are glabrous without macro- and micro-hair. (**B**) The macro-hair density in 370 natural rice populations. (**C**) The micro-hair density in 370 natural rice populations. (**D**) The haplotype of the promoter region of *OsSPL10* (Chr6 27114977) in 12 rice species and their flag leaf trichome phenotype. Species #1, #4, #5, #6, #8, #9, and #10 contain trichome in the flag leaf, while species #2, #3, #7, #11, and #12 do not contain trichome in the flag leaf. (**E**) the model of trichome leaf trichome development.

To evaluate whether the TP-ARMS developed in this study can be effectively applied in practical breeding applications, we applied it to the screening of trichome density-related traits in rice. Trichomes play an important role in rice resistance to pests and diseases. Rice trichome can be divided into three types: macro hairs, micro hairs, and glandular trichome [22]. We focused on the macro hairs and micro hairs, which contribute most substantially to pest resistance. We measured trichome density in 370 natural rice populations and, based on the distribution patterns of macro and micro hairs on the leaf surface, classified the leaves into four distinct types (Figure 5A). Considerable variation in the density of both macro and micro hairs was observed among different rice varieties (Figure 5B,C).

Through GWAS analysis, we identified a major QTL on chromosome 6 that regulates rice trichome development. This QTL was identified to be *OsSPL10*, a gene previously reported to regulate trichome development in rice [23]. We further identified a 6-bp indel at Chr6 27,114,977 in the promoter region of *OsSPL10*, which can be classified into two haplotypes: haplotype GCTCACA and haplotype G. This indel may influence trichome number, as the promoter region plays a crucial role in regulating *OsSPL10* expression [23]. We further performed the analysis on the rice 3K genome website. Interestingly, the major haplotypes of Chr6 27,114,977 in *japonica* and *indica* rice were GCTCACA and G. In *japonica* rice, haplotype GCTCACA accounted for only 4.2%, while haplotype G accounted for 95.8%. In *indica* rice, haplotype GCTCACA accounted for 96.3%, while haplotype G accounted for only 3.4%. This indicates that this indel has a completely opposite distribution in *japonica* and *indica*. This implies that this locus may have an important role in the subsequent breeding process. We then screened our rice germplasm and randomly selected 12 rice accessions, which were genotyped for the GCTCACA and G haplotypes using TP-ARMS. Interestingly, there are 5 species show glabrous leaf phenotype which are haplotype G in Chr6 27,114,977 (Figure 5D, accessions #2, #3, #7, #10, and #12), while other 7 rice species are contains trichome in their leaf (Figure 5, accessions #1, #4, #5, #6, #8, #9, and #11).

Currently, conventional molecular testing relies on standardized DNA extraction and PCR amplification, which is difficult to be widely adopted in resource-limited scenarios. Although several low-cost extraction methods have been reported [24], there is a lack of systematic verification on whether the products are compatible with the equally simplified downstream PCR process. This compatibility gap between the upstream and downstream steps may hinder the true application of integrated simple solutions.

To assess the performance of TP-ARMS using DNA obtained via a low-cost extraction protocol, genomic DNA was isolated from two parental lines and ten F1 individuals using both the standard CTAB method and the rapid extraction protocol described above (Supplementary Figure S3). For each DNA sample, the two TP-ARMS reactions were conducted in parallel, and banding patterns generated from the two extraction methods were directly compared. The TP-ARMS consistently produced clear and readily interpretable allele-specific bands for all samples, irrespective of the DNA extraction method used. Genotype calls were fully concordant between CTAB- and rapid-extracted DNA samples (12/12 samples, 100% concordance). These results demonstrate that the TP-ARMS is tolerant of DNA with moderate purity and is compatible with simple, low-cost extraction procedures commonly employed in resource-limited laboratories.

Using this approach, the TP-ARMS was subsequently applied to genotype natural rice populations exhibiting variation in trichome density (Figure 5E), further supporting its suitability for large-scale breeding and trait-screening applications.

## 3. Discussion

InDel markers have been extensively employed in traditional breeding programs to track agronomically valuable traits, from disease resistance to abiotic stress adaptation [25,26]. However, the utility of large InDels (>20 bp) is constrained by their low abundance in plant genomes, with fewer than 5% of curated InDels exceeding this size in crops [27]. In contrast, small InDels (1–10 bp) are far more abundant and often directly affect phenotypes, as they are located in or near coding and regulatory regions. Despite their functional significance, conventional methods routinely overlook these subtle variations due to technical limitations. Agarose gel electrophoresis, with a resolution limit of approximately 5–10 bp, cannot reliably resolve smaller InDels even with high-concentration gels (4–5%). Polyacrylamide gel electrophoresis (PAGE) can achieve single-base resolution but requires toxic reagents, prolonged run times (>4 h), and specialized imaging equipment. The development of the Tri-Primer Amplification Refractory Mutation System (TP-ARMS) addresses this critical gap, enabling rapid, cost-effective detection of InDels as small as 1 bp—a breakthrough with profound implications for both basic research and applied breeding.

The TP-ARMS achieves unparalleled resolution for small InDels by synergizing allele-specific primer design with a streamlined two-tube workflow. Traditional agarose gel electrophoresis fails to resolve InDels below 5 bp due to its reliance on fragment length separation rather than sequence specificity. Although PAGE-based methods can resolve 1-bp differences, they require toxic acrylamide, precise temperature control, and post-electrophoresis silver staining. These steps make the procedure approximately three times more labor-intensive than the TP-ARMS. By contrast, the TP-ARMS leverages allele-specific 3′ mismatches in forward primers (e.g., insertion-F and deletion-F) to ensure amplification only in perfectly matched templates, enabling discrimination of 1-bp InDels on standard 1% agarose gels. This innovation reduces reagent costs by 40% compared to KASP markers, which require proprietary fluorescent probes priced at $0.50–$1.00 per reaction.

The cost-effectiveness of the TP-ARMS is further underscored by its elimination of specialized equipment. For example, genotyping 100 rice samples for a 3-bp InDel in *OsBph14* costs $12.50 using the TP-ARMS (primers: $10; agarose: $2.50) versus $125 for Sanger sequencing. This 90% cost reduction is transformative for breeding programs in developing regions, where per-project budgets often fall below $5000. Moreover, the TP-ARMS tolerates DNA of moderate quality (A260/A280 ≥ 1.5), unlike KASP assays requiring high-purity DNA (A260/A280 > 1.8), making it ideal for field-collected samples exposed to environmental degradation.

Importantly, the applicability of the TP-ARMS is not restricted to a single species or trait. In quinoa (*Chenopodium quinoa*), the method distinguished parental lines of Faro and QQ65 based on a 5-bp InDel in *CqREBCL1*, a gene regulating epidermal bladder cell (EBC) density—a trait critical for salt tolerance. Similarly, in rice, the TP-ARMS identified a 3-bp InDel in *OsNRAMP5* linked to cadmium (Cd) accumulation, enabling breeders to develop low-Cd varieties that reduce grain Cd levels.

Taken together, these results position the TP-ARMS as an intermediate genotyping strategy that bridges the gap between low-resolution gel-based markers and high-cost fluorescence-based platforms. The TP-ARMS fills a practical gap between conventional gel-based markers (e.g., SSRs and size-based indel assays) and fluorescence-based high-throughput platforms (e.g., KASP). Unlike standard agarose indel markers that rely on fragment length separation, TP-ARMS uses allele-specific 3′ mismatches to discriminate small InDels (1–6 bp) and therefore achieves single-base resolution without PAGE or capillary electrophoresis. Compared to Sanger sequencing, the TP-ARMS is far less expensive and faster for routine genotyping, while sequencing remains the gold standard for definitive base-level confirmation of novel variants. Compared to KASP, the TP-ARMS does not require specialized fluorescent instrumentation or proprietary probes and tolerates DNA of moderate purity. This makes it particularly well suited for marker-assisted selection at small or medium scales in resource-limited settings. Conversely, KASP is advantageous when very large sample numbers and plate-based throughput are needed and when laboratories have access to fluorescence detection equipment and strict sample QC. A concise qualitative comparison of TP-ARMS, Sanger, and KASP is provided in Table 1 to help practitioners choose the most appropriate platform for their context. We note that we did not perform experimental KASP assays in the present study due to lack of access to a fluorescence plate reader in our laboratory; comparisons with KASP in this manuscript are therefore literature-based.

Despite its strengths, the TP-ARMS has two main limitations. First, primer design can be complex. Allele-specific primers require careful optimization of 3′ mismatch positions to balance specificity and amplification efficiency. For example, InDels within homopolymeric regions (e.g., poly-A/T tracts) exhibit <50% primer success rates due to nonspecific binding. Machine learning tools like Primer-BLAST could mitigate this by predicting optimal mismatch configurations. Second, throughput is limited to one locus per reaction. Current workflows process one locus per reaction, whereas multiplexed KASP assays handle 10–20 loci simultaneously. Integrating fluorescent labels (e.g., FAM/HEX) or size-barcoded amplicons (100–500 bp increments) could enable multiplexing without eroding the method’s cost advantages.

## 4. Materials and Methods

### 4.1. Plant Materials and DNA Extraction

To examine the utility of the TRI-ARMS marker we developed, we performed tests using rice and quinoa. Rice and quinoa seeds were washed and placed into 9-cm dishes fitted with filter paper for germination. After 3 days, we transferred the germinated seeds of rice and quinoa to a soil mixture consisting of biomass and vermiculite in a 3:1 ratio. Leaves were collected from individual plants grown in incubators under long-day conditions (16 h light and 8 h dark).

Genomic DNA extraction was performed by homogenizing plant material in liquid nitrogen, followed by rapid extraction of DNA from plants using 1% CTAB. The concentration of extracted DNA was determined by Nanodrop2000 (Thermo Fisher Scientific, Wilmington, DE, USA).

### 4.2. Sequence Alignment and Primer Design

The genes related to Nip and B5 in rice were downloaded directly from the NCBI database. The gene sequences of different quinoa cultivars were obtained from our previous resequencing data. Sequence alignment was performed with the use of Geneious software (version 10.2.6), with forward primers designed for regions in which the sequence was indel and universal reverse primers designed for regions in which the sequence was identical. Primers were generated by Geneious. The generated primers were tested for sequence specificity using primer blast from NCBI.

### 4.3. PCR Amplification and Marker Validation

For PCR analysis, leaves were harvested from rice and quinoa plants for 14 days. Plant leaf tissue DNA was extracted using CTAB. PCR was performed through 35 cycles, including denaturation (95 °C for 20 s), annealing (60 °C for 15 s), and extension (72 °C for 30s). The results were verified by electrophoresis on a 1% agarose gel at 120 V for 20 min. The size of the PCR amplified fragments was determined using DNA marker from the Trans2K^®^ DNA Marker 2kb ladder (TransGen Biotech, Beijing, China).

### 4.4. Rice Trichome Phenotyping

The completely expanded leaves of rice were taken, and 1 mm × 1 mm leaves were cut along the middle vein. The leaves were placed under a microscope (LEICAEZ4W, Leica Microsystems, Wetzlar, Germany) at 30 times magnification for observation, and the supporting software Leica Application Suite V4 was used for image acquisition. The collected pictures were then statistically analyzed in Image J (version 1.8.0) and corrected by naked eye counting (/mm^2^).

### 4.5. GWAS Analysis

Genome-Wide Association Study (GWAS) was performed using the GAPIT R package (R4.2.1) [28]. Statistical significance was determined using the Bonferroni correction threshold (*p* = 1/total SNP number), a widely adopted approach in plant genetic studies.

## 5. Conclusions

The TP-ARMS bridges the resolution-accessibility divide in molecular genotyping, democratizing precision breeding for small InDels. Its simplicity, cost-efficiency, and taxonomic flexibility make it indispensable for both high-tech laboratories and field stations in the developing world. As global agriculture confronts climate change and soil degradation, TP-ARMS-equipped breeders will be uniquely positioned to deploy resilient crop varieties, ensuring food security for future generations.

## Figures and Tables

**Table 1:** Comparison of TP-ARMS with Sanger Sequencing and KASP Technology.

Feature	TP-ARMS (This Study)	Sanger Sequencing	KASP (Competitive Allele-Specific PCR)
Principle	Two-tube allele-specific PCR with shared reverse primer; readout by presence/absence of bands on agarose	Direct sequencing of amplicons (bidirectional)—base-level resolution	Allele-specific PCR with fluorescent probes and endpoint fluorescence clustering
Resolution for small InDels	Effective for 1–6 bp via allele-specific primer discrimination (not dependent on gel length separation)	Gold standard—single bp resolution	Suited for SNPs and small indels (1–6 bp) when assays are properly designed
Accuracy (typical)	High when primers are optimized; validated vs. Sanger in this study for the reported loci	Very high; gold standard for validation	Very high when well optimized; widely used in breeding labs
Throughput per run	Medium (single locus per PCR; many samples possible by plate format and multiple thermocyclers)	Low (per sample sequencing)	High (plate-based 96/384 formats)
Per-sample cost (indicative)	Low (standard PCR + agarose)	Moderate–high (per sample sequencing fees)	Moderate–low at scale; higher up-front assay development cost
Equipment required	Basic thermocycler, agarose gel box and imager	Thermocycler; sequencing can be outsourced	Real-time PCR/fluorescence reader or service provider
DNA quality requirements	Tolerant of moderate purity (works with CTAB and rapid extracts as shown)	Moderate (cleaner templates produce better sequence reads)	Requires consistent, relatively high-quality DNA for reliable clustering
Hands-on time (per locus per ~96 samples)	Moderate (PCR + gel)	Low per PCR; additional time for sequencing prep and turnaround	Low after setup; high throughput reduces per-sample hands-on time
Hazardous reagents	No acrylamide required	Standard PCR reagents	Standard PCR reagents
Multiplexing capacity	Limited (single locus per reaction currently)	Possible by barcoding multiple amplicons	Limited per assay but many assays can be run in parallel
Suitability for field/low-resource labs	High	Moderate (outsourcing possible)	Low–moderate (requires specialized equipment or outsourcing)
Typical failure modes	Primer design failures in difficult sequence contexts (e.g., homopolymers)	Poor reads if PCR fails or template impure	Poor cluster separation with low DNA quality or suboptimal assay
When preferred	Large-scale genotyping of small InDels where low cost and minimal equipment matter	Definitive validation and discovery of novel variants	Large scale genotyping campaigns with access to readers and strict QC

## Data Availability

The original contributions presented in this study are included in the article/Supplementary Materials. Further inquiries can be directed to the corresponding authors.

## Supplementary Materials

Supporting information can be downloaded at the following address: https://www.mdpi.com/article/10.3390/ijms27031406/s1.
