# Supplementary material for: TP-ARMS: A Cost-Effective PCR-Based Genotyping System for Precision Breeding of Small InDels in Crops
Source: Int J Mol Sci. 2026 Jan 30;27(3):1406. doi: 10.3390/ijms27031406 (PMC12898809; doi:10.3390/ijms27031406)
Supplement: Supplementary file 1 [file ijms-27-01406-s001.zip › ijms-4104490-supplementary.pdf]

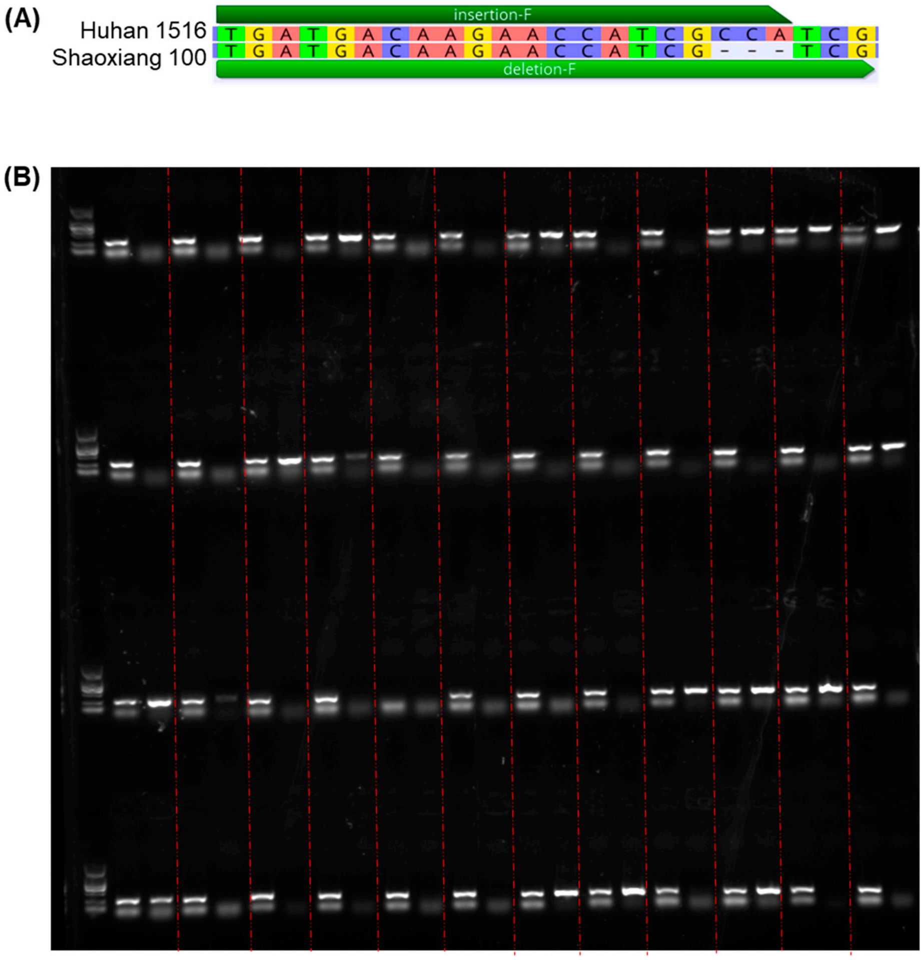

Supplementary Figure S1. TP-ARMS could well identify the BC1F2 population derived from the cross between *Shaoxiang 100* and *Huhan1516*. (A) There was a 3bp indel difference in exon 11 of *OsNRAMP5* between *Huhan1516* and *Shaoxang 100*. (B) The genotype of BC1F2 population can be accurately known by TP-ARMS. Two reactions were required for genotype identification of each individual. The first reaction was to identify whether individuals contained DNA from *Huhan 1516*, while the second reaction was to identify whether individuals contained DNA from *Shaoxiang 100*. After agarose gel electrophoresis detection, we could identify the genotype information of each individual.

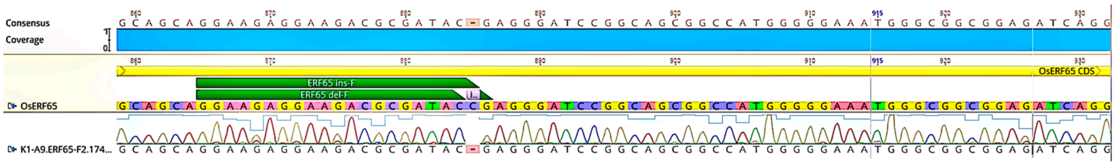

Supplementary Figure S2. Sanger sequencing validation of a 1-bp deletion in the OsERF65 mutant. The sequencing result confirms the frameshift mutation, which was successfully detected by the TP-ARMS assay. This validates the specificity of the TP-ARMS method for distinguishing the mutant allele from the wild-type DNA.

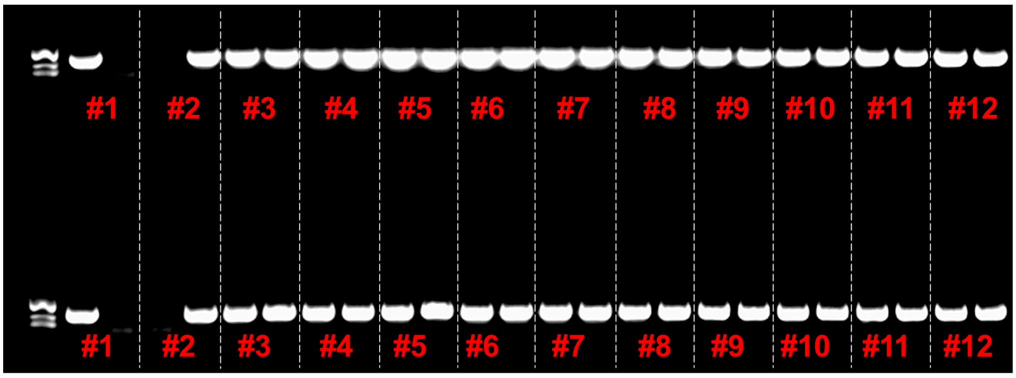

Supplementary Figure S3. Representative agarose gels showing TP-ARMS results for CTAB vs low-cost rapid DNA extraction. DNA was extracted from two parental rice lines (#1 , #2) and ten F1 individuals using CTAB(up panel) and the rapid extraction protocol (down panel) adapted from [24]. For each DNA sample both TP-ARMS reactions (insertion-F + universal R; deletion-F + universal R) were run and loaded side-by-side on the gel. Banding patterns are concordant between extraction methods for all samples (12/12 concordant). Lanes:#1, Parent A; #2, #3-#12 F1-1 to F1-10, F1 individuals.
